# Advances in Accurate Microbial Genome-Editing CRISPR Technologies

**DOI:** 10.4014/jmb.2106.06056

**Published:** 2021-07-09

**Authors:** Ho Joung Lee, Sang Jun Lee

**Affiliations:** Department of Systems Biotechnology, Chung-Ang University, Anseong 17546, Republic of Korea

**Keywords:** CRISPR, Cas9, genome editing, bacteria, mismatch intolerance

## Abstract

Previous studies have modified microbial genomes by introducing gene cassettes containing selectable markers and homologous DNA fragments. However, this requires several steps including homologous recombination and excision of unnecessary DNA regions, such as selectable markers from the modified genome. Further, genomic manipulation often leaves scars and traces that interfere with downstream iterative genome engineering. A decade ago, the CRISPR/Cas system (also known as the bacterial adaptive immune system) revolutionized genome editing technology. Among the various CRISPR nucleases of numerous bacteria and archaea, the Cas9 and Cas12a (Cpf1) systems have been largely adopted for genome editing in all living organisms due to their simplicity, as they consist of a single polypeptide nuclease with a target-recognizing RNA. However, accurate and fine-tuned genome editing remains challenging due to mismatch tolerance and protospacer adjacent motif (PAM)-dependent target recognition. Therefore, this review describes how to overcome the aforementioned hurdles, which especially affect genome editing in higher organisms. Additionally, the biological significance of CRISPR-mediated microbial genome editing is discussed, and future research and development directions are also proposed.

## Introduction

More than three decades ago, Ishino *et al*. discovered repeating sequences with symmetry structures in the 3’-end of the *iap* gene of *Escherichia coli* [1]. Given that sequencing data was far scarcer at that time, the authors could not identify prokaryotic homologous sequences containing the aforementioned repeating sequences, and thus their biological significance remained uncharacterized. Further, Mojica *et al*. identified similar palindrome sequences in the genomes of 20 species of bacteria and archaea. The researchers addressed the need for studies regarding the universality, phylogeny, and biological significance of the repeating sequences [2]. Ruud Jansen and Francisco Mojica referred to this “interspersed short sequence repeat family” as CRISPR (Clustered Regularly Interspaced Short Palindromic Repeats), which is exclusively found in prokaryotes and not in eukaryotes or viruses [3].

More recent studies determined that the spacer DNA sequences in CRISPR are derived from extrachromosomal genetic elements such as bacteriophages and conjugative plasmids, suggesting that CRISPR acts as an adaptive immune system in bacteria [4]. Experiments conducted in 2007 confirmed that CRISPR effectively acts as a bacterial adaptive immune system by correlating the inactivation of the *cas* gene with phage sensitivity in *Streptococcus thermophilus*, an industrial strain common in dairy products [5]. In 2010, the CRISPR/Cas mechanism was elucidated. The cells that survived infections from agents containing exogenous DNA such as phages or plasmids integrated the foreign sequences into their own CRISPR locus. These sequences were subsequently processed into crRNAs as a guide for Cas nucleases and ultimately interfered with future invasions from entities carrying the same nucleic acid sequences [6]. In August 2012, Jennifer Doudna, Emmanuelle Charpentier, and their colleagues identified the underlying mechanism of these phenomena, including the formation of a dual-RNA complex that directs the Cas9 nuclease to the target site, after which the tracrRNA:crRNA-guided Cas9 nuclease can cleave double-stranded DNA targets adjacent to the 5’-GG dinucleotide protospacer adjacent motif (PAM) [7].

The CRISPR/Cas system consists of a target-recognizing RNA and a DNA-cleaving Cas nuclease, which are functionally independent. Therefore, to modify the target DNA sequences, the CRISPR/Cas must only alter the target-binding sequence in the target-recognizing guide RNA [8]. In the CRISPR/Cas9 system, two separate crRNAs and tracrRNAs are linked with a loop to form a chimeric single guide RNA (sgRNA), thus facilitating genome editing [7]. Among the many known Cas nucleases, single-polypeptide Cas9 nuclease is the most widely used [9]. After the *Streptococcus pyogenes*-derived Cas9 nuclease was first used [7], various orthogonal Cas9 nucleases derived from *Staphylococcus aureus* [10], *Streptococcus thermophilus* [11], *Neisseria meningitidis* [12], *Francisella novicida* [13], and *Campylobacter jejuni* [14] were discovered. In vivo genome editing using CRISPR/Cas9 was first reported in human cells [15, 16]. Currently, the CRISPR/Cas9 system has become a major genome editing tool not only in bacteria but also in plants [17], animals [18], and human cells [19] for a variety of applications including industrial processes, agriculture [20], and the development of therapies against human diseases [21]. In this review, we have summarized studies ranging from classical genome engineering methods to more recent CRISPR-mediated genome editing, particularly in bacteria. Moreover, the development of various accurate technologies including single-base level genome editing was described. Finally, we discussed perspectives on the future development of CRISPR technology and basic research on genomics and molecular genetics.

## Bacterial Genome Editing Methods

**Fig. 1:**
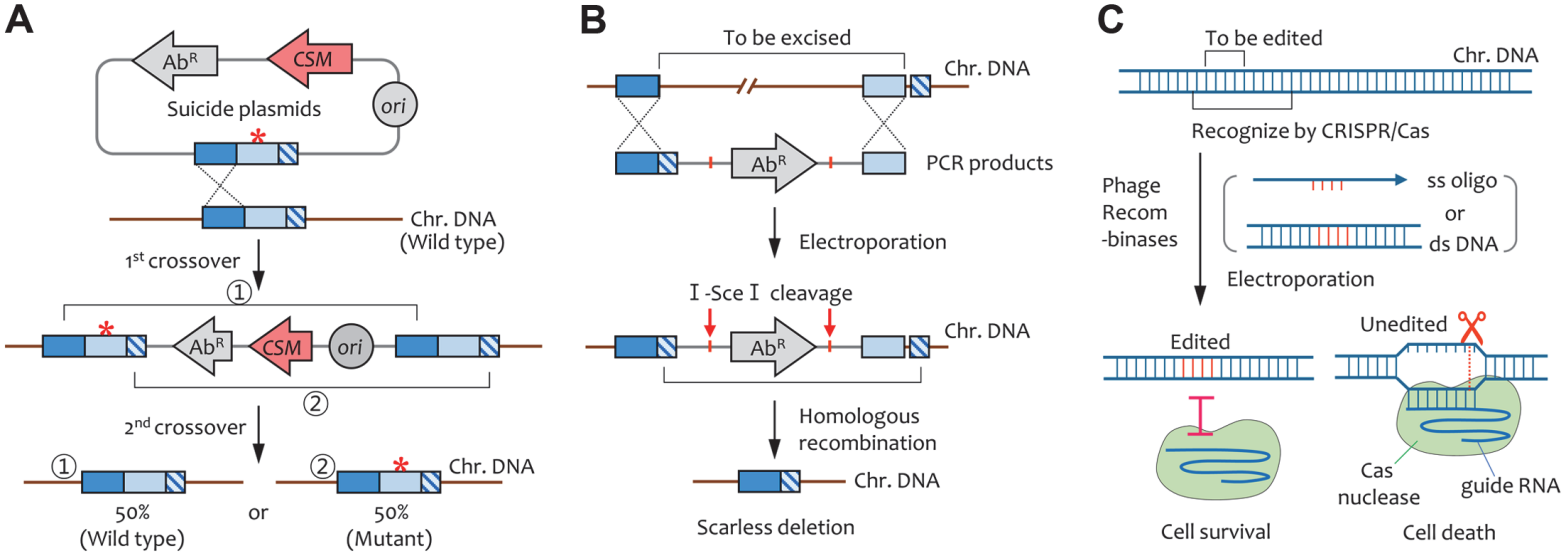
Genome engineering methods in bacteria. (**A**) Genome editing using counter-selectable markers (CSM). The first single crossover results during the integration of suicide plasmids. The second crossover can be selected via counterselection (*e.g.*, sucrose in the media is toxic to cells when the *sacB* gene is present in the genome), which results in the excision of the plasmid backbone. Editing and non-editing events have an equal likelihood of occurrence (*i.e.*, a fifty-fifty chance). (**B**) Scarless gene deletion. Three different homologous DNA fragments were rearranged, and an antibiotic resistance marker (Ab^R^) flanking I-SceI cleavage sites was inserted in the middle of three PCR-fused DNA fragments. The chromosomal DNA sequences to be excised were deleted via HR without any traces or scar sequences after I-SceI cleavage. The dotted lines indicate the homologous recombination between chromosomal DNA and PCR products. Red arrows indicate an I-SceI cleavage. (**C**) CRISPR-mediated genome editing. Phage recombinases facilitate the integration of donor templates (ss oligos or dsDNA) into the chromosomal DNA. Unedited DNA targets are recognized and cleaved by the guide RNA/Cas nuclease complex. Unedited cells die, as most bacteria lack the DSB repair system. Finally, edited cells are obtained via CRISPR/Cas negative selection.

Several bacterial genome editing methods have been described thus far, including CRISPR/Cas technology (Fig. 1). First, the introduction of point mutations into the genome can be achieved using suicide plasmids harboring the mutations flanking homologous DNAs and counter-selectable markers [22]. The presence of DNA fragments including both the desired mutations and counter-selectable markers in the genome is toxic to cells under specific conditions [23]. Plasmid backbones can be eliminated while leaving behind the desired mutations via double-crossover and counter-selection [24].

Second, scarless gene deletion has been achieved by generating double-strand breakages (DSBs) of DNA using meganucleases such as I-*Sce*I, followed by homologous recombination (HR)-mediated DNA repair [25]. Phage-derived recombinases have been recently used to promote HR with donor DNAs such as PCR products and oligonucleotides, followed by CRISPR-mediated negative selection of unedited cells [26].

To edit bacterial genomes, external DNA molecules harboring the desired sequences (plasmids, PCR products, and single-stranded DNA oligos) must be introduced into the cells. The inserted DNAs must have homologous sequences to introduce the desired mutations into the target site via HR. This process can be facilitated by recombinases in the host cells (*e.g.*, RecA in *E. coli*) or derived from bacteriophages (*e.g.*, λ-Red and RecET). λ-red recombinases (Exo, Bet, and Gam) can promote HR events between the target site and the PCR products harboring approximately 50-bp homologous sequences at both ends [27]. For example, the Keio knockout collection (3,985 individual gene knockout mutants of *E. coli*) was constructed using this method [28]. Moreover, effective recombination using mutagenic oligonucleotides as donor DNA can be achieved by co-expressing only the λ-red Bet protein [29].

During genome editing, it is not possible to introduce external donor DNA sequences into the genome of all surviving cells. Therefore, the edited cells where the external DNA sequences were successfully introduced must be easily distinguished. Positive selection can be conducted using non-replicating vectors or PCR products harboring drug-resistance marker genes. Further, second cross-over induction via counter-selection can increase genome editing efficiency (Fig. 1A). The double-crossover method has been used for genome engineering in various bacteria. For example, a suicide vector harboring both the desired mutation and the *sacB* gene was used to edit the genome sequence of *Corynebacterium glutamicum* [24]. A toxic levan is generated by levansucrase (encoded by the *sacB* gene) in the cells in sucrose-containing media [23], which kills cells carrying the plasmid backbone and the *sacB* gene, thus leaving only live cells with the desired mutation via double-crossover. In *Bacillus subtilis*, the *upp* gene (encoding uracil phosphoribosyl-transferase) was used as a counter-selectable marker in 5-fluorouracil containing medium [30].

Genome editing using selectable markers leaves scars on the bacterial chromosome even if the selectable marker genes are removed using flippase (FLP) or Cre recombinase [31, 32]. More importantly, the scar sequences may interrupt the expression of the edited genes and/or downstream iterative editing procedures. To solve these issues, a scarless genome editing method has been developed (Fig. 1B). A linear DNA molecule carrying three different DNA fragments homologous to the genomic DNA sequence and an antibiotic selectable marker flanked by two I-*Sce*I meganuclease sites was generated via overlap PCR. After electroporation and integration of the PCR products, the chromosome was cleaved by I-*Sce*I and the DNA breakage was connected via RecA-mediated DSB repair. Finally, the genomic DNA to be excised was removed without any scars or traces [25].

There are two major DSB repair pathways. One is non-homologous end joining (NHEJ), which can reconnect 3'-hydroxyl and 5'-phosphate without a repair template. The other is homology-directed repair (HDR), which is based on the recombination between two DNA molecules carrying sequences that are homologous to each other [33]. Reportedly, when DSB occurs in higher organisms such as humans, the deacetylation of FOXL2 via translocation of SIRT1 activates and enhances the recruitment of the Ku complex on the DSB site, which subsequently promotes the initiation of NHEJ [34]. In contrast, in the case of prokaryotic systems, NHEJ pathways are only observed in some bacteria such as *Mycobacterium tuberculosis* [35], *M. smegmatis* [36], *Pseudomonas aeruginosa* [37], and *Bacillus subtilis* [38]. Most bacteria rely on HDR primarily when DSBs occur in the cell [39]. Given that cells lacking the NHEJ pathway cannot survive when the genome contains DSBs, the recognition and cleavage of unchanged DNA targets via CRISPR/Cas negative selection has been frequently used for bacterial genome editing. Using this approach, only living cells with the desired donor DNA sequence mutation are preserved, whereas those without the mutation are eliminated (Fig. 1C) [40].

## CRISPR-Mediated Genome Editing

The potential applicability of CRISPR/Cas9 as a genome-editing tool based on in vivo cleavage activity [7] has allowed for genetic modification in living organisms [15, 16]. Various novel genome editing techniques were later developed, including gene disruption using a drug marker [26], gene deletion [41], and point mutation [42]. In *Streptomyces* sp., gene deletion was performed using a single plasmid harboring the cas9 gene, sgRNA, and donor DNA with homologous arms [43]. CRISPR/Cas9 system has been also used for engineering of secondary metabolite biosynthetic gene clusters in *Streptomyces* [44]. A method to achieve seamless genomic DNA deletions or insertions was established in *Lactococcus lactis*, which exceeded a 75% editing efficiency within 72 h [45]. Plasmid-based gene integration and deletion were achieved in *Pseudomonas putida* within 5 days using a plasmid that harbored a homolog sequence and I-*Sce*I sites [46]. A double gene deletion method was developed in *Streptomyces* species using a single plasmid [47]. Further, four *E. coli* loci were simultaneously edited with a >30% efficiency using three plasmids harboring λ-red recombinases, multiplex donor DNA, and multiplex gRNA, respectively [48]. However, the excision or insertion of several-kilobase sequences may affect the physiology of the modified organism [49] or lead to genome instability [50]. Therefore, these challenges highlight the need for precise and scarless genome editing capable of modifying only a few essential bases.

Representative case studies on CRISPR-mediated pinpoint microbial genome editing are summarized in Table 1. Base-editings by CRISPR scissors were screened and confirmed by phenotypic changes in *C. glutamicum* [51] and *Synechococcus* UTEX 2973 [52], respectively. PAM-containing sequences were edited in *Bacillus subtilis* [53] and *Lactobacillus luteri* [54]. The co-expression of bacteriophage λ derived red-recombinase highly improved the genome editing efficiency of CRISPR/Cas9-mediated negative selection [26, 55-57]. Another Rac prophage-derived RecT-mediated ssDNA recombineering approach also improved genome editing efficiency in *C. glutamicum* [51] and *Lactobacillus plantarum* [58]. Impairing the interaction between primase and replisome boosts ssDNA loading on the replication fork, thus improving recombination efficiency in the *dnaG* mutant of *E. coli* [59]. In *Clostridium beijerinckii*, a two-step single nucleotide modification method was established using artificial PAM or protospacers [60]. Similarly, alterations in the original protospacer achieved using artificial sequences were reverted back to the original sequences via point mutation [61].

Various CRISPR systems have been discovered since their discovery and widespread adoption, which are generally divided into two classes and six types. Among them, the CRISPR/Cas12a system has a single polypeptide nuclease similar to Cas9, which can only induce DSBs in the target DNA when a crRNA is present. Therefore, both CRISPR/Cas12a and CRISPR/Cas9 have similar performances and applications [62]. This system is now generally referred to as Cpf1 (CRISPR from *Prevotella* and *Francisella* 1). Using a *C. glutamicum* industrial strain, 2 bp substitutions were introduced by oligonucleotide-directed mutagenesis and Cpf1 negative selection with a nearly 100% editing efficiency [63]. Similarly, 2 to 3 bp substitutions were also introduced in *E. coli*, Yersinia pestis, and *Mycobacterium smegmatis* with editing efficiencies of up to 90% [55].

## PAM-Independent Genome Editing

In 2008, researchers from a dairy company first discovered that there is a specific sequence of DNA motifs that are adjacent to the protospacer in the phage sequence, which corresponded with the newly acquired spacer in the *Streptococcus thermophilus* strain [64]. Later, Francisco Mojica and colleagues coined the term “protospacer adjacent motif ” (PAM) to refer to this DNA motif [65]. There are dozens of known PAM sequences in Cas9 orthologs [66]. PAMs play an important role in the recognition and selection of the protospacer during spacer acquisition [67]. The CRISPR locus of host cells lacks the PAM to escape autoimmunity from the Cas nucleases, and therefore the Cas nucleases are allowed to discriminate between the self- and non-self-DNA [68]. However, the specific sequence requirements limit the available targeting ranges [69].

According to the crystal structure of the Cas9-sgRNA-DNA ternary structure, it was found that 1097-1368 amino acid residues in the C-terminal region of SpCas9 constitute the PAM-interacting (PI) domain [70]. Since it was likely that PI domain determines the PAM specificity, protein engineering approaches were performed to alter the PAM specificity. Cas9 variants with altered PAM specificities were obtained through bacterial selection-based directed evolution [71]. Meanwhile, Cpf1 variants with modified PAMs were obtained by performing a structure-based mutagenesis to increase the DNA targeting range of Cpf1 [72]. Recently, a phage-assisted continuous evolution method developed Cas9 variants with expanded PAM sequences [73]. In addition, a Cas9 variant that recognizes the 5'-NG sequence as PAM, so called Cas9-NG, was created through rational design based on structural information [74].

Cas9-NG significantly reduces PAM restrictions, as it only requires a single nucleotide (G). PAM specificity can be indirectly defined through the binding affinity and repression of the target gene using catalytically deactivated Cas9 (dCas9) and dCas9-NG. Previous studies have demonstrated that dCas9 and dCas9-NG are less restricted by PAM requirements, suggesting that target binding and target cleavage are mediated by different mechanisms [75, 76].

## Single-Base Genome Editing

**Fig. 2:**
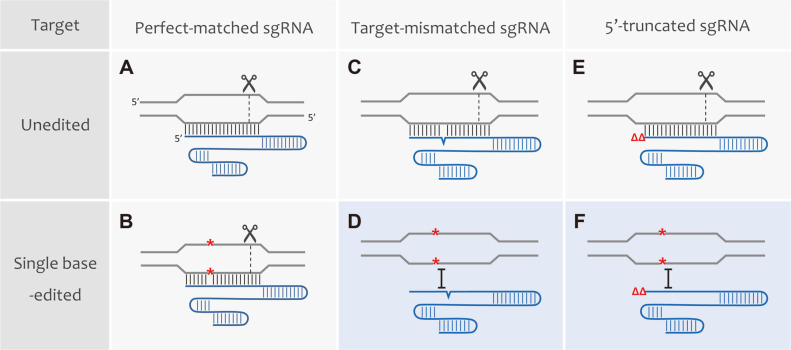
CRISPR-mediated bacterial single base genome editing by target-mismatched and 5’-truncated sgRNAs. (**A**) Unedited target DNAs are cleaved by the gRNA/Cas9 complex. (**B**) Single base edited cells are also cleaved by the gRNA/Cas9 complex due to mismatch tolerance. Unedited target DNAs are still cleaved by the (**C**) target-mismatched or (**E**) 5’-truncated sgRNA/Cas9 complex. Mismatch intolerance of (**D**) target-mismatched or (**F**) 5’-truncated sgRNA/Cas9 complex enables bacterial single base genome editing (blue-shaded panels).

The CRISPR/Cas system must recognize and cleave foreign DNA to ensure cell survival even if a mutation occurs in the phage genome protospacer or the spacer sequences in the CRISPR loci. Therefore, CRISPR/Cas systems have evolved to allow a certain level of homology [77]. By allowing several mutations, the 1~2 bp edited target DNAs are also recognized and cleaved (Figs. 2A, 2B, 2C, and 2E) [78], which is referred to as mismatch tolerance. Additionally, another study demonstrated the unintended cleavage of DNAs harboring several mismatches with guide RNA [79]. These off-target effects negatively affect the accuracy of genome editing [80]. Unwanted DNA cleavages of the off-target are highly toxic and could induce cell death [81]. Therefore, several strategies to minimize off-target effects were developed to increase on-target specificity or prevent the formation of DSBs using modified Cas nuclease, particularly in higher organisms.

Engineered base deaminases were fused with dCas9 [82], dCpf1 [83], or Cas9 nickase (nCas9) [84] to introduce single base edits without DSBs (base editor). However, given that the linker provides flexibility to the base editor, the unwanted bystander base within the editing window could also be edited [85]. To overcome this bystander effect, the editing window range can be widened or narrowed by altering the fused base deaminase [86]. A base editor using cytidine deaminase was used in *Pseudomonas putida* for multiplex single base editing [87]. Additionally, the engineered reverse transcriptase was fused with nCas9 to synthesize a new DNA strand from the RNA editing template extended from gRNA (prime editor) [88]. In theory, a prime editor can be used to introduce all types of genome alterations [89]. However, this approach cannot be easily implemented in bacterial systems due to the large size of engineered protein constructs [90]. Further, prime editing may introduce small insertions beyond the template primer due to reverse-transcriptase-mediated extension [8].

Guide RNAs (gRNAs) have also been modified to enhance the accuracy and editing efficiency of the CRISPR/Cas genetic scissors. It is reported that the 5’-end truncation of sgRNA increases the target specificity of the sgRNA/Cas9 complex [91] and also enables allele-specific genome editing in mammalian cells [92]. It has also been reported that crRNA extension of Cpf1 [93], chemical base modification of crRNA [94], and 3’-end uridylation of crRNA [95] improved editing efficiency. Furthermore, the mismatch(es) between the target recognition sequence of sgRNA and the target could decrease off-target effects [96].

The use of mismatch intolerance successfully enabled single base editing in bacteria through gRNA modification. In *E. coli*, successful single base editing with up to 95% editing efficiency has been reported using target-mismatched sgRNAs harboring mismatched sequence(s) coupled with unchanged target DNAs (Fig. 2D)[97]. The design rules for mismatched sgRNAs were optimized in 16 randomly chosen genome-wide targets. Target-mismatched crRNA also increased the single base editing efficiency of RecT-mediated oligonucleotide-directed mutagenesis followed by Cpf1 negative selection to up to 99% in *C. glutamicum* [98]. Additionally, the 5’-end truncation of sgRNA can facilitate single base editing in bacteria (Fig. 2F) [99]. The 5’-truncated sgRNA method can be used to edit each base in the target region using the same plasmid regardless of the edited locations and mutation types within the target region. Using this approach, single-base genome editing can be achieved more easily and accurately.

CRISPR/Cas genetic scissors are well suited to edit bacterial genomes, as will be described below. Bacterial genome sizes are generally below 4^12^ bp. Therefore, the likelihood that a particular sequence of more than 12 bp occurs at different locations of a bacterial genome is extremely low. In other words, off-target effects are not a concern, as these are observed only in a few species. The consequences of off-target effects would be negligible in bacterial systems because they are present at the population level. Furthermore, the implementation of mismatch intolerance facilitates adequate base editing efficiency in bacterial genomes. Since the target specificity and editing efficiency were relatively high, the target-mismatched or 5’-truncated sgRNA method can be expanded to mammalian genome editing.

## Perspectives

The CRISPR/Cas system was proposed as a genome-editing tool over a decade ago, and research continues to explore new methods for applying this technology to all living systems, from bacteria to humans. Particularly, bacterial systems have been used in the development and validation of new functions or modifications of the CRISPR/Cas system due to their convenience and higher productivity. In many cases, bacterial systems have been used for the development and improvement of Cas nuclease variants. Engineered Cas9 nucleases with reduced PAM dependence [73, 74], base editors [82-84], and prime editors [88] have been created using bacterial and(or) bacteriophage systems that will continue to be used in the future.

**Fig. 3:**
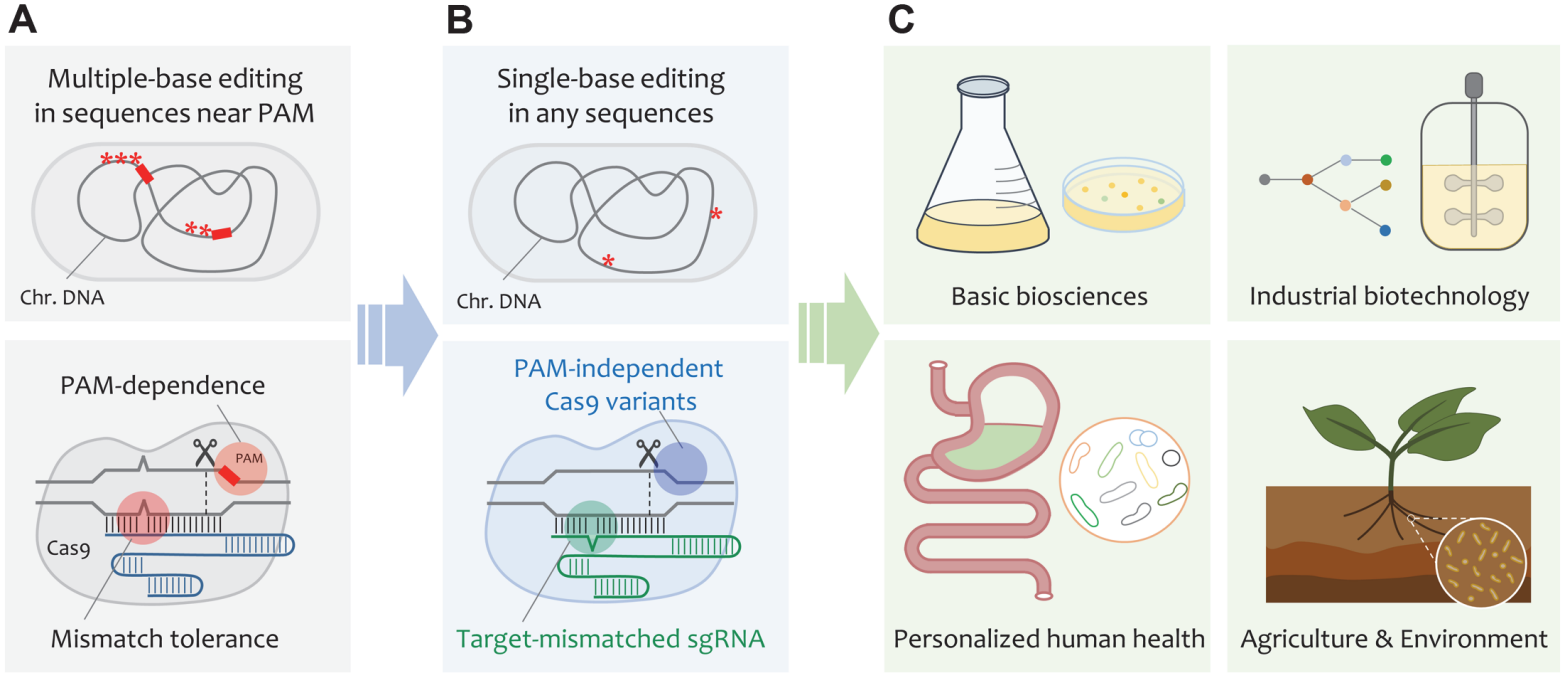
Development and application of CRISPR-mediated microbial genome editing tools. (**A**) Major limitations of accurate CRISPR technologies: PAM-dependence, and mismatch tolerance. Red rectangles, and asterisks represent PAM sequences and mutated bases in the genome. (**B**) Development of single-base level CRISPR genome editing methods by target-mismatched sgRNAs and engineered Cas9 variants. Red asterisks represent single point mutations. (**C**) Application of microbial CRISPR genome editing in various fields: Basic biosciences (*e.g.*, DNA metabolism in model microbes); Industrial biotechnology (*e.g.*, engineering of microbial workhorses); Personalized human health (*e.g.*, modification of gut microbiome); Agriculture and environment (*e.g.*, regulation of plant-microbes interaction).

Even a single base or nucleotide alteration in the genome can result in important phenotypic changes. Accurately modifying single bases is critical to induce the desired phenotype or revert a mutant sequence to its wild-type state. In terms of genome editing, there is no single method that serves all specific purposes. For example, methods based on dCas9 variants cannot introduce or edit indel mutations in the genome, and therefore the Cas9 nuclease must be used instead. Therefore, accurate nucleotide editing approaches that overcome mismatch tolerance and/or off-target effects are of paramount importance for the improvement of CRISPR/Cas9 nuclease-mediated genome editing methods. Therefore, future research should focus on the development of highly accurate CRISPR technologies in bacteria and their application to higher organisms. The development and application of cutting-edge CRISPR genome editing tools in the field of microbial biotechnology is illustrated in Fig. 3.

CRISPR/Cas9 systems can cause DNA damage such as double or single-strand breaks at specific locations in the genome, thus providing a model system for the study of DNA repair or recombination. NHEJ may easily cause indel mutations that can modify the open reading frame, thus resulting in gene dysfunction. Recently, the accuracy of NHEJ was analyzed to elucidate the mechanism of DNA repair on DSBs generated by CRISPR/Cas9 [100]. The target gene can then be edited as desired by introducing donor DNA via HDR. However, current research has not yet determined whether donor DNA is incorporated after DNA target cleavage or whether it is negatively selected by Cas9 cleavage after donor DNA incorporation. This phenomenon may be closely related to the mechanisms of DNA repair, recombination, and replication in cells. Prokaryotic genome editing using CRISPR/Cas could serve as a good model system to provide insights into the molecular mechanisms of DNA metabolism.

With current CRISPR/Cas9 genome editing methods, bacterial cells with the desired mutation can be obtained in less than a week. The development of more accurate and faster genome editing tools (*e.g.*, single-base level and multiplex editing) will enable the improvement of bacterial strains with industrial applicability within a few days. In the future, the technological convergence of CRISPR/Cas and -omics tools applicable to various bacteria will allow for the modification of entire microbiomes to deliver personalized health care.

## Figures and Tables

**Table 1:** CRISPR/Cas-mediated pinpoint microbial base editing.

Edited length	Efficiency (%)	Cas system	Donor DNA	Organism	Target gene	Description	Reference
Single base	99.7	FnCpf1	Oligo	*Corynebacterium glutamicum*	*crtEb*	Target-mismatched crRNA	[98]
	95	SpCas9	Oligo	*Escherichia coli*	*galK*	Target-mismatched sgRNAs	[97]
	83	SpCas9	Oligo	*E. coli*	*galK, xylB*	5’-truncated sgRNAs	[99]
	68	SpdCas9-CBE	-	*Agrobacterium tumefaciens*	*atu1060*	Curing of the plasmid using *sacB* counter-selection	[101]
	65	SpCas9	Oligo	*E. coli*	*rpsL*	Single base substitution for streptomycin resistance acquisition	[42]
	30	SpCas9	Plasmid	*Clostridium beijerinckii*	*pta*	Two-step single nucleotide modification using artificial PAM or two-step indel	[60]
Double bases	100	SpCas9	dsOligo	*Schizosaccharomyces pombe*	*swi6*	Short-homology-mediated genome editing	[102]
	100	SpCas9	Oligo	*Lactobacillus plantarum*	*rpoB*	RecT-aided ssDNA recombineering	[58]
	100	AsCpf1	Oligo	*C. glutamicum*	*crtYf*	Comparison of 7 different Cas system	[103]
	100	FnCpf1	Oligo, PCR products	*C. glutamicum*	*argR*	RecT-aided ssDNA recombineering	[63]
	100	FnCpf1	Oligo	*Zymomonas mobilis*	*ldh*	Plasmid curing system using its native plasmid	[104]
	90	BbCpf1	Oligo	*C. glutamicum*	*crtYf*	Comparison of 7 different Cas system	[103]
	87.5	FnCpf1	Plasmid	*E. coli*	*prpC*	λ-red aided two-plasmid system	[56]
	76	FnCpf1	Oligo	*E. coli*	*lacZ*	Using X-Gal for blue-white colony screening	[55]
	60	FnCpf1	Oligo	*Mycobacterium smegmatis*	*ms1521*	λ-red aided ssDNA recombineering	[55]
	60	TsCpf1	Oligo	*C. glutamicum*	*crtYf*	Comparison of 7 different Cas system	[103]
Triple bases	100	SpCas9	Plasmid	*Bacillus subtilis*	*trpC*	Substitution of PAM sequence	[53]
	100	SpCas9	Oligo	*Streptococcus pneumoniae*	*bgaA*	Amino acid change and creating BtgZⅠ site	[42]
	94 ~ 99	SpCas9	Oligo, PCR products	*E. coli*	*rpoB, ack*	λ-red aided ssDNA recombineering	[57]
	70 ~ 86.7	SpCas9	Oligo	*C. glutamicum*	*rpsL, rfp*	Editing of streptomycin resistance phenotype or integrated fluorescence gene	[51]
	71	SpCas9	Oligo	*L. plantarum*	*glmS1*	Overexpression of Dam to interfere MMR	[105]
Quadruple bases	47	FnCpf1	Oligo	*C. glutamicum*	*mscCG*	Site-directed mutagenesis using a random mutagenic oligo	[106]
	17	Synthetic Cas9	PCR products	*Ustilago maydis*	*cdk5*	Introduction of point mutations using two-step PCR with three DNA fragments	[107]
